# Functionally Generated Amalgam Stops for Single Complete Denture: A Case Report

**Published:** 2009

**Authors:** Pravinkumar G. Patil, Rambhau D. Parkhedkar

**Affiliations:** * Lecturer, Department of Prosthodontics, Government Dental College and Hospital, Nagpur, India; ** Professor, Department of Prosthodontics, Government Dental College and Hospital, Nagpur, India

**Keywords:** Amalgam, balanced dental occlusion, complete denture

## Abstract

Single complete denture opposing natural dentition is a common occurrence in clinical practice. This article reports a case of a single complete denture with a technique of occlusal refinement by functionally generated amalgam stops condensed in prepared resin teeth after initial balancing of the denture with semi-adjustable articulator. This technique provides intimacy of contact in all excursions by carving the amalgam in plastic stage. Amalgam stops improve the efficiency of the resin teeth. Dentures fabricated using this technique require fewer and simpler post-insertion adjustments.

## Introduction

Single complete denture (SCD) opposing natural dentition is a common occurrence in clinical practice. Malposed, tipped or supraerupted teeth in the opposing arch is a perplexing problem in achieving a harmonious balanced occlusion in SCD patients.^1^ Balanced occlusion is a bilateral, simultaneous, anterior, and posterior occlusal contact of teeth in centric and eccentric positions.^2^ It is developed for stability of denture bases in relation to supporting structures during functional and parafunctional movements.^3^–^5^ Lack of occlusal balance may lead to denture instability, mucosal soreness, tissue changes and accelerated ridge resorption. Various techniques have been described to achieve a balanced occlusion in SCD patients.^1^,^6^–^9^ It falls into two categories as follows: 1) those that dynamically equilibrate the occlusion by the use of a functionally generated path and 2) those that statistically equilibrate the occlusion using an articulator programmed to simulate the patient’s jaw movement. The functionally generated chewin techniques seem to provide the most accurate method of recording occlusal patterns.^1^ Another problem with SCD is attrition of denture teeth if resin teeth are used and attrition of natural teeth if porcelain is used. In this situation, gold occlusals, interpenetrating polymer network (IPN) and resin teeth with amalgam stops have been tried as occlusal posterior tooth forms.^1^,^9^ Amalgam stops wear less and cause less wear of opposing natural teeth.^10^ Amalgam is less expensive than porcelain, IPN or gold occlusals. Also, amalgam stops have been described as improving the efficiency of the resin teeth.^10^ This article describes a technique of occlusal refinement by functionally generated amalgam stops after initial balancing of SCD with articulator generated pathway.

## Case Report

**Figure 1:**
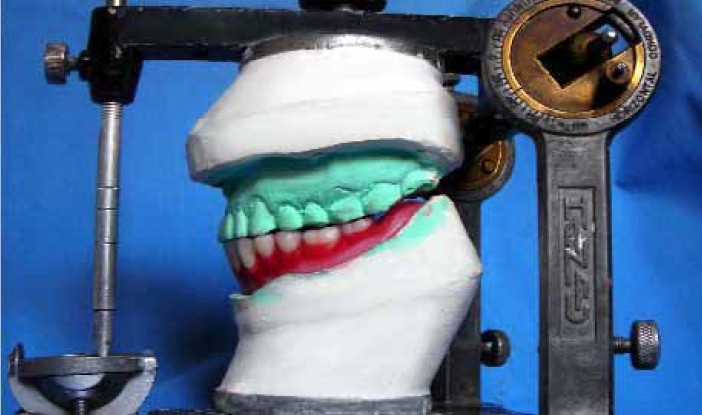
Use of programmed articulator to achieve balanced occlusion with mandibular trial SCD.

**Figure 2:**
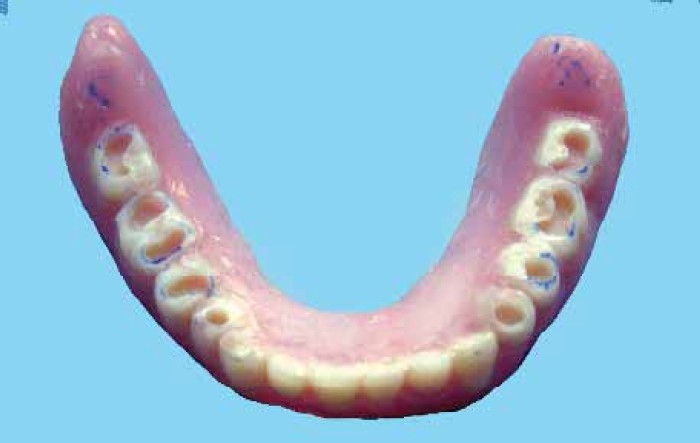
Marking centric and eccentric records

**Figure 3:**
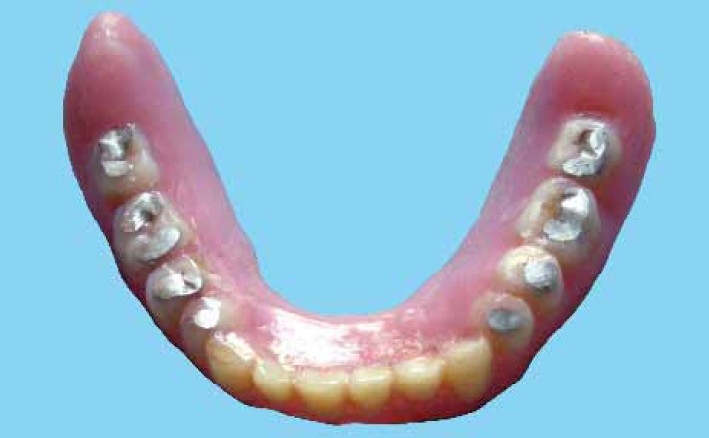
Occlusal preparations (note borders of the tracing areas are spared)

**Figure 4:**
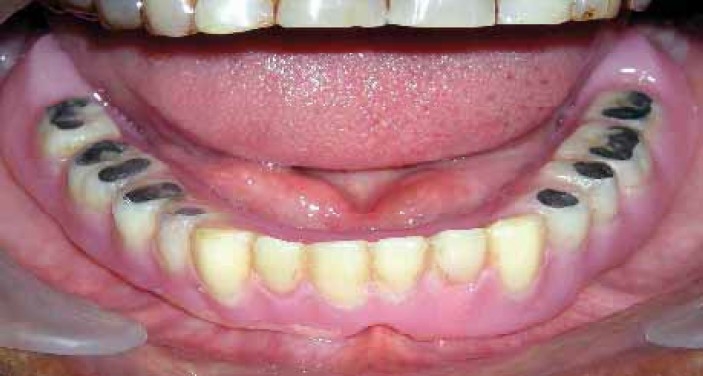
Final functionally generated occlusal pattern developed in amalgam

**Figure 5:**
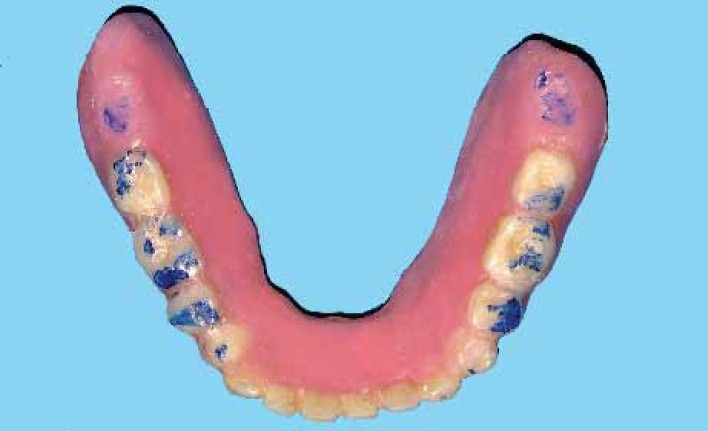
Single complete denture in use

**Figure 6:**
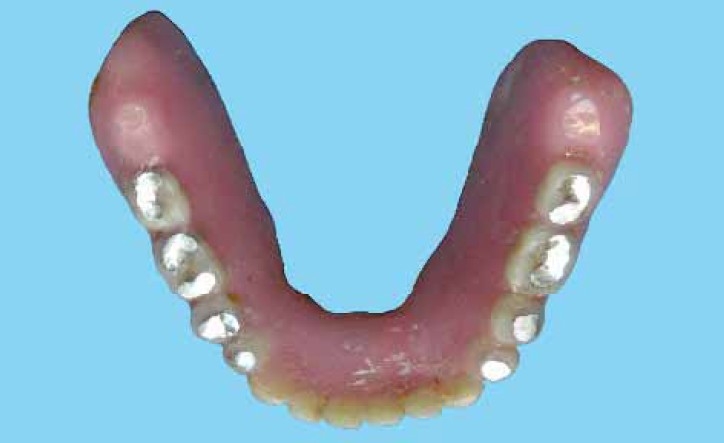
Occlusal view of the SCD after two years.

**Figure 7:**
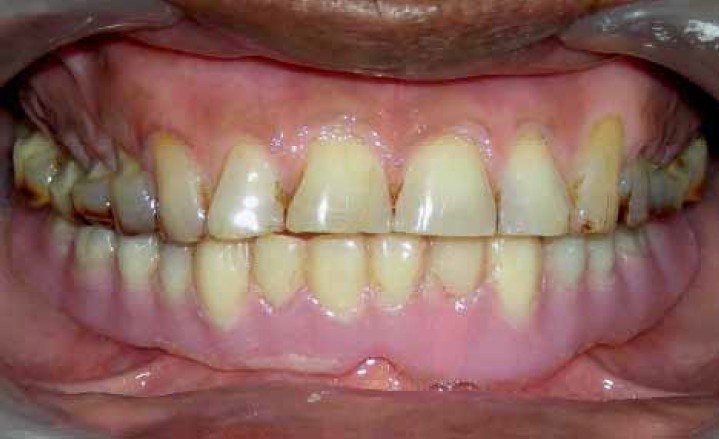
Final functionally generated occlusal pattern developed in amalgam

A 65 year-old male patient was presented in the department of prosthodontics with a completely edentulous mandibular arch and completely dentulous maxillary arch. A SCD was fabricated in the usual manner.^1^ A balanced occlusion was achieved with acrylic resin teeth (Acryrock, Ruthinium Group Dental Manufacturing, Badia Polesine, Italy) using semi-adjustable articulator (Figure 1).^1^,^9^ At the denture delivery appointment, a new centric relation interocclusal record was made and occlusal adjustments were carried out by remounting the processed SCD on the semi-adjustable articulator. Occlusal equilibration was also achieved in patient’s mouth by selective grinding procedure. Centric as well as eccentric occlusal contacts were marked intraorally with the help of articulating paper by asking the patient to perform all functional jaw movements (Figure 2). Occlusal preparations (1.5-2 mm in depth) were carried out in premolar and molar denture teeth, using inverted cone diamond bur extending to include as much of the articulating paper tracing as possible and sparing thin borders of the tracing areas to maintain vertical dimension (Figure 3). A mix of amalgam was triturated according to the manufacturer’s directions. Amalgam was then condensed into the occlusal preparations with the help of amalgam condenser. Preparation should be overpacked 1 mm or more using heavy pressure to ensure margins completely covered with well-condensed amalgam.^11^ Denture was placed into patient’s mouth and the patient was directed to close with tapping action on the teeth in centric relation and to perform all eccentric movements in order to carve the condensed amalgam (Figure 4). In this situation, the vertical relation at occlusion was maintained by unprepared borders of tracing areas. Excess amalgam removed with the help of high volume evacuator. Patient was instructed to continue all functional movements of the jaw until initial setting of the amalgam occurred. Excess amalgam was removed again and occlusal pattern was examined for deficient margins. The denture was delivered (Figure 5) and postinsertion instructions were given. The patient was recalled after 24 hours. Amalgam stops were polished by an amalgam polishing kit.^11^ The patient was followed up at periodic recall appointments and the last recall visit was after 2 years. The amalgam occlusal stops had a highly smooth and shiny surface (Figure 6). There was no noticeable clinical evidence of enamel wear of opposing natural teeth. The loss of vertical dimension was clinically insignificant. The patient used the denture with great functional comfort for 2 years (Figure 7).

## Discussion

The major disadvantage of acrylic teeth used in SCD opposing natural dentition is rapid wear on occlusal surfaces. This affects the vertical dimension of occlusion and tooth relationships, and results in increased horizontal stresses and their associated sequelae.^12^ The rate of wear also depends on the patient’s functional and parafunctional habits. Inserting amalgam stops into the resin occlusal surfaces slows down and controls this wear.^1^,^9^,^10^

This article describes a case report of a SCD patient with a technique of occlusal refinement by functionally generated amalgam stops condensed in prepared resin teeth after initial balancing of the denture with semi-adjustable articulator. The amalgam stops can be carved either on the programmed articulator or directly in the patient’s mouth. The amalgam stops generated on the programmed articulator are carved according to the articulator movements guided by condylar and incisal guidance. The programmed articulator cannot perfectly copy patient’s mandibular movements as there may be a positive or negative error in the articulator adjustment.^13^ Also, one or more mechanical limitations of the articulators like straight condylar path, fixed intercondylar distance, mechanical incisal guide table, or accuracy and stability of eccentric records prevent exact simulation of mandibular movements.^13^ Hence, the balanced occlusion (achieved with articulator-generated amalgam stops) ultimately needed to be refined in patient’s mouth by selective grinding procedure in the end. The functionally generated path technique described in this article carves the amalgam in plastic stage directly in the patient’s mouth. Thus, the intimacy of contact in all excursion is possible and need not to be refined by selective grinding procedure (which is required in articulator generated path technique). TThis technique is less time consuming and more accurate than articulator-generated amalgam stop technique. But, if the denture bases do not have intraoral stability or if the patient is physically unable to form a chew-in record, the articulator-generated path technique will be preferred. The occlusion developed with a functionally generated amalgam stop technique provides a simple and inexpensive procedure to establish a balanced occlusion with extended wear capabilities. The functionally generated path in amalgam insert is in harmony with the patient’s mandibular movements and is an exceptionally smooth, efficient and comfortable balanced occlusion. Dentures fabricated using this technique require fewer and simpler postinsertion adjustments. The only disadvantage using amalgam stops (either articulator or functionally generated) is their esthetic unacceptance.
